# Dose determination of sufentanil for intravenous patient-controlled analgesia with background infusion in abdominal surgeries: A random study

**DOI:** 10.1371/journal.pone.0205959

**Published:** 2018-10-17

**Authors:** Luming Zhen, Xiao Li, Xue Gao, Haidong Wei, Xiaoming Lei

**Affiliations:** Department of Anesthesiology, The Second Affiliated Hospital of Xi’an Jiaotong University, Xi’an, Shaanxi, China; National Taiwan University, school of dentistry, TAIWAN

## Abstract

**Objectives:**

Sufentanil has been widely used in epidural PCA, while its use in intravenous PCA has rarely been reported. Based on its use in target controlled infusion, we reckoned that the effect-site concentration of sufentanil would be steady if background infusion is given in intravenous PCA. This prospective, single center, randomized study with a three arm parallel group design aims to find out the appropriate dose of sufentanil when used in intravenous PCA with background infusion in abdominal surgeries.

**Methods:**

Patients diagnosed with gastrointestinal cancer and consented to the study were recruited. The analgesia pump with one of three different doses of sufentanil (1.5, 2.0 or 2.5 μg/kg) was attached to the patient through peripheral venous line right after surgery. The primary endpoint was pain scale VAS up to 48 hours postoperatively.

**Results:**

In our study 90 patients were analyzed. In group B (SF 2.0) and C (SF2.5), patients had better pain relief than in group A (SF 1.5). There was no difference between group B and C in pain intensity at rest. While in group C more patients got pain relived at activity than in group B. All three groups had low and similar incidence of adverse effects of sufentanil.

**Conclusion:**

The dose 2.5 μg/kg of sufentanil with background infusion is preferred because of better pain alleviation at activity without increase of adverse effects up to 48 hours after surgery.

## Introduction

Many patients experienced acute postoperative pain, and approximately 86% had moderate, severe, or extreme pain. Although pain management system has been fully established in most institutions, yet there were still many patients complaining of inadequate pain control after acute pain care [1], which would affect quality of life, functional recovery, and increase risks of complications and postoperative chronic pain [2].

Sufentanil, one of the opioids with strong analgesic potency, is now widely used for surgery patients during anesthesia, has also been extensively used in postoperative pain control and labor pain relief, specifically in patient controlled epidural analgesia (PCEA) [3–5]. Although PCEA is preferred in certain types of surgery than patient controlled intravenous analgesia (PCIA) because of better pain relief and less side effects [6], but no long-termed outcome was superior to PCIA [7]. Besides to the association to postoperative hypotension and delay of urinary catheter removal, actually, epidural analgesia is technically difficult in certain cases and even contradicted to some cases. Abundant observations on the PCEA using sufentanil combined with local anesthetics have been conducted [8], but there are very few reports about PCIA using sufentanil. With appropriate administration regimen, sufentanil PCIA would achieve great satisfaction like other opioids. Inspired by the use of sufentanil in target controlled infusion (TCI) [9], we proposed that intravenous PCA with continuous background infusion would have a steady effect-site concentration compared to intravenous PCA with only PCA bolus dose which could cause fluctuation of plasma concentration. Sufentanil TCI consists of a priming dose, a decreasing infusing rate and the constant maintaining rate. Then, a loading dose followed by a constant continuous infusion could mimic the TCI regimen of sufentanil. Moreover, the PCA bolus dose with constant background infusion could only cause slight fluctuation of the effect-site concentration of sufentanil. Therefore, we designed this study to determine if there was a certain dose of sufentanil better than other doses in relieving postoperative pain using PCIA in patients underwent moderate surgeries. The primary endpoint of the study was pain scale VAS.

## Methods

### Study design

This is a prospective, single center, randomized study with a three arm parallel group design. No changes were made to methods after trial commencement.

### Ethical approval

This trial was registered at ClinicalTrials.gov (NCT02503826). The study was conducted in accordance with the principles of Good Clinical Practice and the Declaration of Helsinki, approved by the Ethics Committee of the Second Affiliated Hospital of Xi’an Jiaotong University, Xi’an, China (NO. 005 2015, Chairperson Prof. Yang Dechang) on February 5, 2015. Written informed consent was obtained from all patients or a legal surrogate. It was a single center, randomized, double-blind, three arm parallel group study. It was conducted at the Second Affiliated Hospital of Xi’an Jiaotong University between February 11, 2015 and December 8, 2016. The authors confirm that all ongoing and related trials for this drug/intervention are registered. The delay in registering this study is due to: a. failed contact to the organization's PRS administrator to request a user login to the website for the registration (we requested repeatedly but had no reply), b. we had to finally apply a PRS account in person, c. we were not very familiar with the registration process on the website.

### Patient population

Patients diagnosed with gastrointestinal cancer, aging from 20 to 75, BMI 18 to 28 kg/m^2, American Society of Anesthesiologists (ASA) grade I to II, anticipated surgery duration within 4 hours and agreed to sign the consent paper were scheduled for elective abdominal surgery including gastrectomy, colectomy, and rectectomy. Women participants in the study had to either be menopause or use routine contraceptive method.

Patients with known allergy or contradiction to the treatment drugs, or severe respiratory, cardiovascular or neurological diseases, hepatic or renal dysfunction, psychiatric history or unstable mental state were excluded from the study. Patients with history of drug or alcohol abuse, chronic use of opioids and pregnancy or breast-feeding were excluded as well.

### Conduct of the study

Patients were enrolled according to the inclusion and exclusion criteria by our nurse. All patients received general anesthesia. No premedication was given. Ten minutes before anesthesia induction a loading dose of dexmedetomidine with 1 μg/kg was infused over 10 minutes. Then anesthesia was induced with midazolam 2 mg, sufentanil 0.5 μg/kg, propofol 1–2 mg/kg. Cisatracurium 0.2 mg/kg was given to facilitate orotracheal intubation with a cuffed tube. Anesthesia was maintained with continuous infusion of propofol 3–7 mg/kg/h, remifentanil 0.1–0.25 μg/kg/min, cisatracurium 0.1 mg/kg/h and dexmedetomidine 0.2–0.5 μg/kg/h with positive pressure ventilation in a circle system. And the bispectral index (BIS) value was maintained between 40 and 60. Mean blood pressure (MAP), Heart rate (HR), pulse oxymetry (SpO_2_), end-tidal CO_2_ (ETCO_2_), blood loss, and transfusion were recorded during anesthesia. Cisatracurium and dexmedetomidine were discontinued until peritoneum closure, while propofol and remifentanil were stopped until the last stitch of skin.

Patients were enrolled to our study only when the surgery duration was within 4 hours. The eligible patients were randomly assigned to one of three treatment arms (A, B, C). The randomization was generated by permuted block randomization with block size 6 manually. Briefly, there should be 2 patients assigned to A treatment, 2 assigned to B treatment and 2 assigned to C treatment in each block. Fifteen permutations and combinations were selected by a statistician based on random number table. The allocation sequence of patients was determined by the sequence of surgery. The allocation marks were sealed in 90 sequentially numbered envelopes and could only be opened 30 min before the end of surgery by the nurse who was responsible for the preparation of the pump. After checking up with the pump preparation and allocation mark, the allocation mark was concealed in envelop again by the nurse. And the allocation mark remained sealed till complete of last follow-up and start of data input. No changes were made for blinding.

The PCA pump was prepared with different doses of sufentanil and 10 mg tropisetron. The dose per body weight of sufentanil in the intravenous PCA pump was determined by which treatment arm the patient was allocated to, with 1.5 μg/kg sufentanil in arm A, 2.0 μg/kg sufentanil in arm B and 2.5 μg/kg sufentanil in arm C. The total volume of the pump was 100 ml. Immediately after surgery, the PCA pump was attached to the peripheral venous line by the investigator who was unaware of the formula of the PCA pump or the treatment arm. A loading dose of 10 μg sufentanil was administered right at the end of the surgery. And then 5 μg at a time according to the patients’ complaint of pain untill the pain relieved or the respiratory rate (RR) was less than 10 breaths per minute. The emergence and extubation time was recorded and emergence time was defined as the time from anesthesia discontinuation to eye opening on command. The background infusion of PCIA was set at 2 ml/h, the bolus volume of each PCA press was 0.5 ml and lockout interval was 10 min. All patients were monitored in the post-anesthesia care unit (PACU) for at least 30 min until the discharge criteria were met. All the patients were followed-up 2, 6, 24 and 48 hours after the surgery. Clinical evaluations including MAP, HR, RR, SpO_2_, total pressing times, total consumption of sufentanil, rescue analgesic requirement, visual analogue scale (VAS), numerical rating scale (NRS) and Ramsay sedation scale (RSS) score were recorded. Side effects including nausea and vomiting, urinary retention, pruritus, respiratory depression were also recorded. Overall satisfaction index of the patients was recorded as well.

The overall study duration of each patient was less than 5 days, including the screening period (1 or 2 days before surgery), the treatment period (from surgery day to 48 hours after surgery) and the following-up period (from the end of the surgery to 48 hours after surgery).

Patients, anesthesiologist, and data collectors involved in the study were unaware of which treatment arm the patients would be in. The double blind method was secured by putting no tag on the PCIA pump.

Rescue analgesic of 5 μg sufentanil was administered intravenously by the investigators when the patient had maximal pressing times per unit time and the pain score was still above 5 at rest for more than 30 min.

The efficacy of the PCIA pump was assessed based on the patients’ pain level of VAS and NRS at the time points of 2, 6, 24, 48 hours after surgery. VAS: the patient was presented with a horizontal 10-cm line (from 0 = no pain to 10 = worst pain) and was asked to mark the spot which indicated the current pain level. NRS: the patient was asked to describe the current pain level through an 11 points numerical scale from 0 = no pain to 10 = worst possible pain.

The safety of the PCIA pump was evaluated based on the incidence, intensity, seriousness, and causality of pump-related adverse events (AEs) and the frequency of clinically significant changes in physical examination, HR, BP, laboratory safety tests (hematology, biochemistry, and urinalysis), and 5-lead ECG.

### Outcomes

The primary endpoint was pain level assessed by VAS up to 48 hours after surgery. Secondary endpoints included NRS score of pain, nausea and vomiting, degree of sedation, hypotension, pruritus, urinary retention, respiratory depression and rescue analgesia. No changes were made to trial outcomes after the trial commenced.

### Statistics

In order to find out the appropriate dose of sufentanil in PCIA after moderate surgery, the primary endpoint pain level assessed by VAS was analyzed. The expected standard deviation of means was 6.9 mm [10], and standard deviation of subjects was 20 mm [11]. The significance level was set at 0.05 and the power at 0.8. Then sample size assumption was made through the software program *PASS 11* (NCSS, LLC, Kaysville, Utah, USA) based on one-way ANOVA. And the calculated sample size was 29 in each group and we included 30 patients in each group. Comparisons of VAS or NRS for pain were performed by linear mixed model among groups using IBM SPSS Statistics 19.0. Ranked data of sedation degree and vomiting and nausea scale were analyzed with the Kruskal–Wallis tests. Probability values under 0.05 were considered significant. No interim analysis was planned and conducted.

## Results

### The participant flow

**Fig 1:**
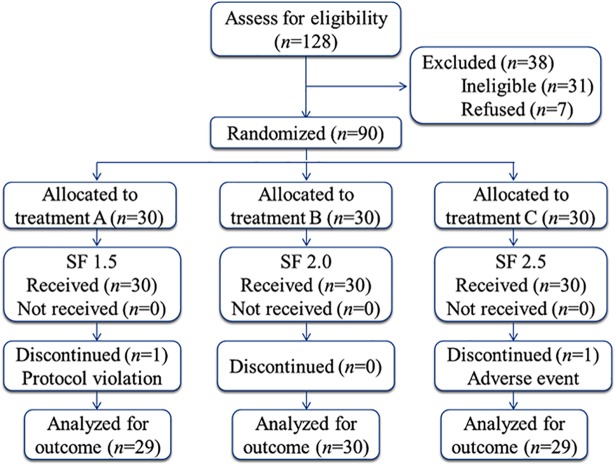
Study CONSORT flow diagram. Patients who were randomly assigned and received the intended treatment were analyzed for the outcomes. Two patients were discontinued: one was discontinued at 38 hours after surgery due to a severe anaphylactic reaction caused by medication treatment in surgical ward, and the other was discontinued at 30 hours after surgery due to violation of the protocol. SF 1.5, 1.5 μg/kg total dose of sufentanil in the PCA pump. SF 2.0, 2.0 μg/kg total dose of sufentanil in the pump. SF 2.5, 2.5 μg/kg total dose of sufentanil in the pump.

Ninety patients in our hospital were assigned to three treatment arms (Table 1). The enrollment started on 11 February 2015 and completed on 5 December 2016, and the last follow-up was on 8 December 2016. The participants received the intended treatment with the randomized group tag (A, B, or C) by time sequence and were analyzed for the primary outcome (Fig 1). Patient data was collected in the operation room and in ward.

**Table 1:** Treatment arms.

Assignment	group	Preparations for PCA pump
A	SF 1.5	sufentanil 1.5 μg/kg, 10 mg tropesitron, diluted to 100 ml
B	SF 2.0	sutentanil 2.0 μg/kg, 10 mg tropesitron, diluted to 100 ml
C	SF 2.5	sufentanil 2.5 μg/kg, 10 mg tropesitron, diluted to 100 ml

### Patient demographics

The mean age of all patients was 60 (range 26–75) yrs old. There were 30 females and 60 males. Patient characteristics and baseline data were compared and no difference among three treatment arms (Table 2). Other related factors like anesthesia duration, surgery duration, surgical types, dose of sufentanil for induction, remifentanil and dexmedetomidine consumptions were comparable among arms as well (Table 2).

**Table 2:** Patients’ baseline characteristics and operational data.

Group		SF 1.5	SF 2.0	SF 2.5
Overall participants		30	30	30
Age, years		60 (10)	61 (9)	60 (10)
Gender, Female/Male		11/19	10/20	9/21
Height, cm		164.4 (7.06)	165.3 (7.40)	165.7 (7.61)
Weight, kg		63.2 (7.90)	64.7 (9.30)	62.45 (10.3)
Hemoglobin, g/L		125.8 (15.09)	124.4 (14.76)	123.8 (21.17)
Creatinine, μmol/l		68.87 (12.39)	65.76 (10.78)	65.49 (11.32)
WBC Count, 10^9 cells/L		6.00 (1.99)	5.53 (1.25)	6.38 (1.98)
Alanine Transaminase, u/l		15.0 (8.49)	18.3 (9.56)	18.5 (12.71)
Blood Urea Nitrogen, mmol/l		4.87 (1.10)	5.63 (1.59)	5.00 (1.91)
Blood glucose, mmol/l		5.0 (0.75)	4.9 (0.66)	4.9 (0.75)
Anesthesia duration, minutes		173 (29.6)	167 (39.0)	182 (39.5)
Surgery duration, minutes		143 (33.3)	140(37.2)	150 (39.2)
Emergence time,minutes		8.6 (3.55)	9.9 (5.56)	10.6 (6.85)
Time to extubation, minutes		12.2 (4.00)	14.1 (7.68)	13.9 (7.09)
Preoperative SBP, mmHg		145(19)	143 (18)	143 (20)
Preoperative HR, beats/min		78 (14)	78 (14)	79 (13)
Remifentanil consumption, mg		1.44 (0.46)	1.41 (0.57)	1.50 (0.58)
DEX consumption, μg		81 (25)	74 (27)	80 (26)
Surgery type, participants				
	stomach	21	17	16
	colon	5	8	9
	rectum	4	5	5

All data is presented as Mean (Standard Deviation) except for gender and surgery types. WBC, White Blood Cell. SBP, systolic blood pressure. HR, heart rate. DEX, dexmedetomidine.

1. The postoperative pain assessment

**Fig 2:**
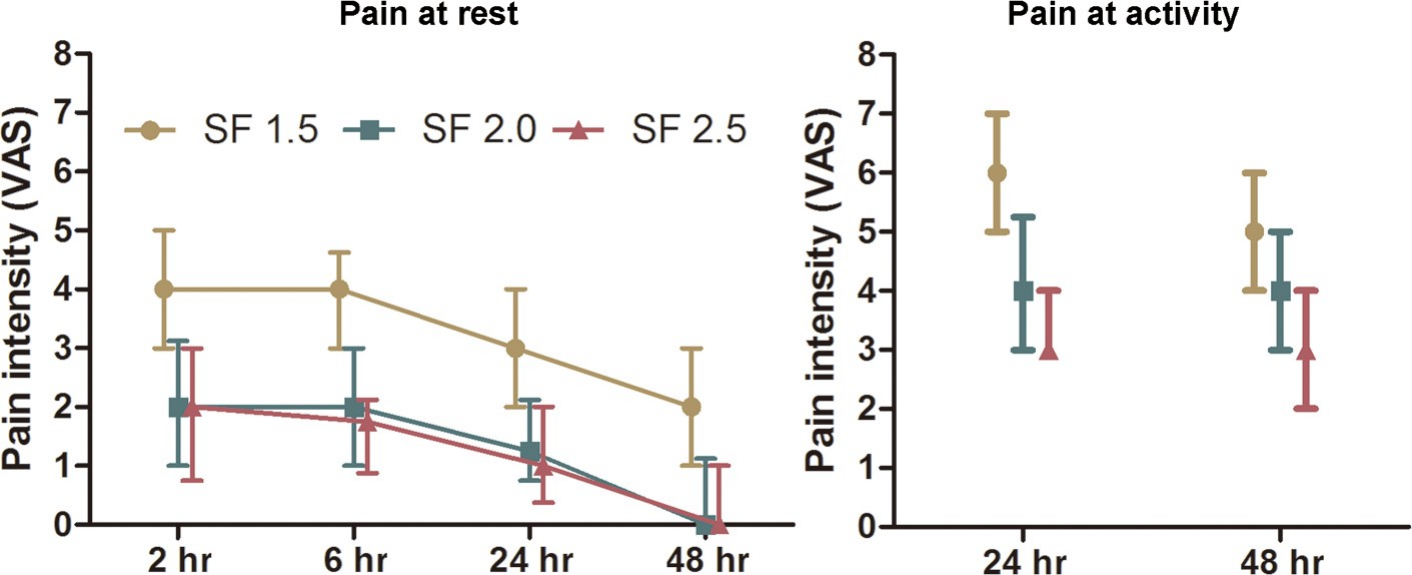
The pain intensity over the first 48 h based on VAS. On the left is the pain intensity at rest over observational time. On the right is the pain intensity when coughing at 24 h and 48 h. The data was presented with median and interquartile range.

**Fig 3:**
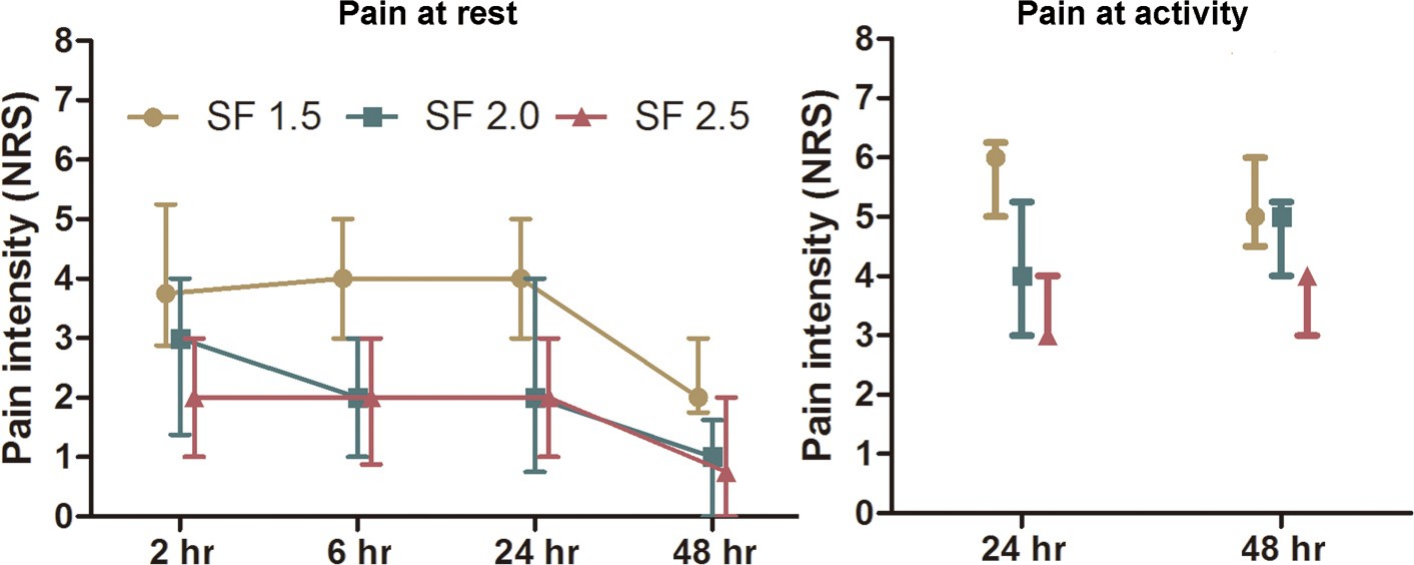
The pain intensity over the first 48 h based on NRS. On the left is the pain intensity at rest over observational time. On the right is the pain intensity when coughing at 24 h and 48 h. The data was presented with median and interquartile range.

The postoperative pain of VAS and NRS was analyzed by mixed linear model. The included factor was group, subjects was set as random effect, repeated effect was time and covariance structure was unstructured. The fixed effect of group for VAS at rest was significant (*P*<0.001), as well as the time effect (*P* = 0.001) and the interaction effect (*P =* 0.033). VAS scores at rest in both group SF 2.0 and SF 2.5 were decreased compared with group SF 1.5 respectively (Fig 2, *P*<0.001). But no difference between group SF 2.0 and group SF 2.5 was detected (*P* = 0.392). The fixed effect of group for VAS at activity was significant (*P*<0.001), as well as the time effect (*P* = 0.001). But no interaction effect was detected (*P =* 0.114). VAS at activity was decreased in group SF 2.0 compared with group SF 1.5 (Fig 2, *P*<0.001). And VAS at activity in group SF 2.5 was decreased as compared with group SF 2.0 (Fig 2, *P* = 0.038). The fixed effects of group for NRS at rest was significant (*P*<0.001), as well as the time effect (*P*<0.001), but not the interaction effect (*P* = 0.138). NRS scores at rest in both group SF 2.0 and SF 2.5 were decreased compared with group SF 1.5 respectively (Fig 3, *P*<0.001), while there was no difference between group SF 2.0 and SF 2.5 (*P* = 0.694). The fixed effect of group for NRS at activity was significant (*P*<0.001) as well as the interaction effect (*P* = 0.028). But no time effect was detected (*P =* 0.980). NRS scores at activity in both group SF 2.0 and SF 2.5 were decreased compared with group SF 1.5 respectively (Fig 3, *P*<0.001). But no difference between group SF 2.0 and group SF 2.5 was detected (*P* = 0.214). In order to further clarify the difference between group SF 2.5 and SF 2.0, we analyzed patients with VAS or NRS scores at activity less than 5.0 [12], and it turned out that in group SF 2.5 there were more patients with VAS or NRS scores at activity less than 5.0 than that in group SF 2.0 at 24 hours after surgery (Table 3).

**Table 3:** Pain score at activity less than 5.0 at 24 hours after surgery.

****Group****	Pain scale at activity less than 5 at 24 hours, participants	
	VAS	NRS
SF 2.0	17/30	18/30
SF 2.5	25/29	25/29
*P* value	0.0078	0.0391

2. The rescue analgesia requests

The rescue analgesia demanding was less in arm B and C when compared with arm A. It was measured by the number of patients who asked for rescue analgesia and the total times of rescue analgesia for each patient (Table 4).

**Table 4:** Rescue analgesia among arms.

Group	Participants	times
SF 1.5	13/30	17/60
SF 2.0	5/30*	6/60*
SF 2.0	3/30*	4/60*

* *P*<0.05 compared with SF 1.5.

3. The opioid related side effects

The number of patients with opioid-related sedation after surgery showed no difference among arms (S1 Table). Patients with RSS scores less than 5 were mentioned in the data and no patient required rescue medication for sedation.

Postoperative nausea and vomiting was documented as well. The number of patients suffering from nausea was no different among arms (S1 Table). No patient suffered from postoperative vomiting in this study.

No respiratory depression was observed, which was defined as respiratory rate less than 10 or SPO_2_ less than 92% when breathing air (S1 Table).

Other side effects related to sufentanil including dizziness, pruritus, headache and constipation were not observed.

## Discussion

In this study we reported the use of sufentanil in postoperative intravenous patient controlled analgesia (PCIA) with continuous background infusion for the first time. Meanwhile we explored the optimal dose of sufentanil for PCIA in abdominal surgeries from three incremental doses.

As to PCA pump parameters, we set background infusion rate at 2 ml/h, bolus volume at 0.5 ml and the lockout time at 10 min. The dose of sufentanil in PCA pump was calculated by the body weight of the patient and the parameters were set based on the sufentanil TCI system of Gepts [13]. After infusion of 10 μg sufentanil, also known as the loading dose, the effect-site concentration would reach to 0.2 ng/l in 6.5 minutes and drop to 0.05 ng/L about 0.5 h later. While the effect-site concentration 0.05 ng/l could be achieved by constantly infusing sufentanil at 0.05 μg/kg/h, which happened to be the background infusion rate in arm C. Each bolus volume of 0.5 ml in arm C would increase the effect-site concentration by 0.01 ng/l within 5 minutes.

Background infusion is not recommended when morphine or fentanyl is used in PCA pump because it won’t improve pain relief and may increase the risk of delayed respiratory depression. The latter is mostly due to the accumulation of opioids during continuous infusion [14,15]. And M6G, the active metabolites of morphine, is partly responsible for respiratory depression caused by morphine [16]. Sufentanil infusion could be adequately described by a linear three-compartmental mammillary model which makes it eligible for target controlled infusion with stable effect-site concentration [13,17]. A reliable target controlled infusion regimen contains a priming dose, a decreasing infusing rate and a constant maintaining rate [17]. Given the linear pharmacokinetics of sufentanil [13], minimal active metabolites with no clinical relevance [18] and its use in target controlled infusion (TCI), the background infusion of sufentanil in PCIA will produce steady effect-site concentration which might effectively alleviate pain with less side effects. In this study, only a few side effects were observed and this might attribute to the steady plasma concentration of sufentanil. Moreover, no severe side effects of sufentanil occurred even in geratic patients (20 of 90 participants older than 70 yr, 6/8/6 in each arm respectively, S2 Table). No deep sedation with the Ramsay scale score less than 4 occurred, no respiratory depression and no vomiting. Nausea was only observed in a few cases. And the low incidence of postoperative nausea and vomiting in this study might attribute to tropisetron prophylaxis during anesthesia and a large dose of tropisetron in PCIA pump formula [19].

It has been reported that the wake-up concentration of sufentanil is around 0.1 ng/ml or higher [20]. Using the TCI system of Gepts, we reckoned that the effect-site concentration of sufentanil in arm C was around 0.05–0.11 ng/ml, which could be achieved with a constant infusion rate of 0.05–0.175 μg/kg/h. Other researchers have observed that patients can be well sedated with sufentanil and midazolam in ICU. When a large dose of sufentanil combined with midazolam was administered, the plasma concentration of sufentanil would be at least 0.3 ng/ml at which patients were still arousable [21]. And in prolonged TCI for postoperative analgesia after cardiac surgery, the concentration of sufentanil was around 0.08–0.1 ng/ml [22]. With lower plasma concentration of 0.05–0.11 ng/ml in arm C in this study, side effects like postoperative sedation might be less than in the studies mentioned above in which the concentration of sufentanil was higher. It might explain why no patients had deep sedation and no sedation needed antagonist in this study. However, this estimation in the study needs to be confirmed by more solid evidence with evaluation of the effect-site concentration.

A report about the comparison of continuous infusion and bolus administration of sufentanil during anesthesia for cardiac valve surgery showed that there was no benefit in continuous infusion except for the simplicity in clinical practice. The continuous infusion group consumed 26% more of sufentanil than bolus administration, while both groups had stable hemodynamics [23]. In our study, background infusion might consume more sufentanil when compared with bolus only. But this needs to be verified because with very large dose of sufentanil administered after cardiac valve surgery, the difference between continuous infusion and bolus might be more significant. Moreover, other evidence showed the combination of TCI and PCA using hydromorphone could provide satisfied postoperative pain relief [24]. With our study we have shown that in simulation of TCI, sufentanil PCIA with background infusion can be regarded as an effective alternative for postoperative pain control.

Even though there was no difference in pain relief at rest in arm B and arm C, we considered that dose 2.5 μg/kg would be the preferred optimal dose. On one hand with 2.5 μg/kg sufentanil more patients experienced pain relief when they were at activity (pain intensity score less than 5 when coughing) 24 hours after surgery. On the other hand, with 2.5 μg/kg sufentanil it didn’t increase side effects compared to 2 μg/kg sufentanil. Theoretically, a higher dose of sufentanil will lead to more significant pain relief because sufentanil has no ceiling effect, as long as the side effects of sufentanil are acceptable. In our study the patients were encouraged to walk in the ward after abdominal surgery as early as possible, and most patients could manage to walk at about 24 hours postoperatively. Therefore, we only observed the pain intensity at activity 24 hours and 48 hours after surgery.

The efficacy of sufentanil PCIA was proved and the safety of its use was preliminarily testified. However, how sufentanil would influence the recovery of patients after 48 hours was not discussed in this study, including physical recovery, the gastrointestinal function and hospital stays. Another question was whether the development of chronic postoperative pain would be influenced by sufentanil PCIA.

Finally, in light of sufentanil TCI we found that sufentanil PCIA with continuous background infusion could provide effective pain relief with less side effects. The optimal dose of sufentanil was 2.5 μg/kg in this study, which is more preferred to the other two lower doses.

This study has several limitations. For a dose-effect study, 30 subjects each group is enough. But if patient safety is to be evaluated, a study with more cases and more centers might be more convincing. The biases of the study were inevitable. The sample size calculation was conducted by a software program *PASS 11* on basis of one-way ANOVA but not the linear mixed model. This would cause incorrect power. We run the linear mixed model analysis and both the effects of treatment and time were significant except for the interaction. Other biases may come from the education backgrounds, personalities and pain sensitivities of all subjects.

## Supporting information

S1 File CONSORT checklist of the study. (DOC)

S2 File English version of study protocol. (DOCX)

S3 File Original version of study protocol. (DOC)

S4 File Raw data files. (RAR)

S1 Table Side effects related to postoperative analgesia among arms. (DOC)

S2 Table Age distribution of old patients. (DOC)
