# Supplementary material for: Dose determination of sufentanil for intravenous patient-controlled analgesia with background infusion in abdominal surgeries: A random study
Source: PLoS One. 2018 Oct 17;13(10):e0205959. doi: 10.1371/journal.pone.0205959 (PMC6192643; doi:10.1371/journal.pone.0205959)
Supplement: S2 File — (DOCX) [file pone.0205959.s002.docx]

Study protocol

The efficacy and dose optimization of sufentanil postoperative patient controlled analgesia after moderate surgery: a single center, randomized, double-blinded trial

Study site: Xi’an Jiaotong University, Second Affiliated Hospital

Responsible Department: department of anesthesiology

Supervisor: Luming Zhen, Haidong Wei

1. Study background

As the “comfort medicine” being developed, pain control rises as a focused issue between patients and physicians. The relief of acute postoperative pain is emphasized among anesthesiologists and surgeons [1-4]. Surgeries injured local tissue, leading to release of histamine and inflammatory factor like bradykinin, prostaglandins, serotonin, and neurotrophic factors etc., which result in the activation of the peripheral nociceptive receptor. The nociceptive signal is transmitted through A δ or C fibers to the dorsal horn of spinal. After serials of complicated modulation, the nociceptive signal is sensed by brain. If the acute pain was not treated properly and in time, the sympathetic nerve system would be stimulated, resulting in increased oxygen consumption of the myocardium, decreased oxygen supply to the myocardium, ultimately increase of perioperative heart ischemia or myocardial infarction. Other harm due to the high tense of sympathetic system is that the recovery of the gastrointestinal function would be delayed, even ileus. Inadequate treatment of the postoperative pain will result in shallow rapid respiration, reluctant to coughing, and eventually increase of the pulmonary complications. It is recognized that lack of postoperative pain control will lead to some undesired influences like the clinic experience of the patients, postoperative rehabilitation, prolonging of the hospital stay, increase of probability of readmission, or even the increased risk of cerebrovascular and cardiovascular events or deaths [5-7]. Besides, if the acute pain was not treated effectively and properly, not only it would severely influence the quality of life, a huge mental distress, but also might develop to chronic pain or neuropathic pain. And it was observed that after surgery, the degree of pain was related to subsequently developed chronic pain [8]. Therefore, in the daily practice o f anesthesia, pain free becomes as our main pursuit.

The management of postoperative pain consists of multiple models and methods. The old ways relied on the intramuscular injection of analgesics by nurses. While the recent decades, the patient controlled analgesia had shown plenty of advantages like effective pain relief, better medical benefits, the participation of patients and satisfaction. The patient controlled intravenous analgesia (PCIA) as one of the main parts of self-analgesia has now become very prevalent during the postoperative period. In the past morphine or fentanyl was used as the main drug for pain control in PCIA. But these two narcotics are inappropriate for continuously infusion because of the pharmacokinetics which might lead to accumulation of drugs and more side effects. And patients would be reluctant to press if the pain was not well relieved. Sufentanil is one of new narcotics deprived of morphine with stronger efficacy and longer duration of effect time. Sufentanil has more stable pharmacokinetics than morphine or fentanyl and more suitable for long duration infusion. Moreover, it has multiple administration routine like intravenous, intranasal, sublingual administration, all of which had been shown safe and effective in postoperative pain control [9-11]. But there are still some deficiencies in the use of sufentanil for postoperative pain like inadequate analgesia which patients complaining of unbearable pain and demanding more analgesics, or over analgesia which results in over sedation or respiratory depression. The dose and efficacy of sufentanil in PCIA is unclear and a proper reference of sufentanil in PCIA is still lacking. Therefore, its very necessary to assess its analgesic effect and ultimately to optimize the use of sufentanil in PCIA. And we conduct this study to observe the use of sufentanil in PCIA after moderate surgery, and to determine the optimal dose of sufentanil with best pain relief and minimal side effects.

1. Study objective

Assess of the analgesic effect of sufentanil in postoperative PCIA and its dose optimization.

1. Study design

A single centered, prospective, randomized, double-blinded trial

1. Study drug

Sufentanil citrate injection, 1ml:50μg, Yichang Humanwell Pharmaceutical Co., Ltd.

Electronic infusion pump, 100ml, Royal Fornia Medical

1. Randomization

The patients are eligible to be randomized provided the surgery duration within 4 hours. The eligible patients will be assigned to one of three treatment arms (A, B, C) through the table of random numbers based block randomization stratified by enrollment sequence. Each block contains 6 patients and there must be 2 patients in each block assigned to A treatment, 2 assigned to B treatment and 2 assigned to C treatment. Fifteen groups of permutation and combinations were combined based on the table of random numbers generated by a statistician. The allocation sequence is according to the sequence of surgery.

1. Blind

The allocation mark is sealed in a sequentially numbered envelope one each till 30 min before the end of surgery. The envelope will be then unsealed by the nurse who is responsible for the preparation of the pump. The observer is another investigator who is blind to the allocation. The nurse and observer must not communicate about the patient’s medication or analgesic effect. The pump has no label on except the enrollment sequence number.

1. Patient population

The patients with no gender limit aging from 20 to 75, BMI 18 to 28 kg/m^2, American Society of Anesthesiologists (ASA) grade I to II, anticipated surgery duration within 4 hours and agreed to sign consent paper are scheduled for selective moderate surgery including abdominal surgery (gastrectomy, colectomy, and rectectomy for cancer). Women participants in the study have to be menopause or use routine contraceptive method.

Patients with known allergy or contradiction to the treatment drugs, or severe respiratory, cardiovascular or neurological disease, hepatic or renal dysfunction, psychiatric history or unstable mental state are excluded from the study. Patients with a history of drug or alcohol abuse, chronic use of opioids and pregnant or breast-feeding will be excluded as well.

1. Study group

The participants are randomly allocated to three groups as following: low dose group (sufentanil 1.5μg/kg), moderate dose group (sufentanil 2.0μg/kg) and high dose group (sufentanil 2.5μg/kg)

1. Anesthesia

Preoperative

All the patients are fasting for 8 hours before surgery and no preoperative medication. After admission in to the operation room, the patient will be supplied with mask oxygen. Then the peripheral venous access will be established. Ringer’s solution will be dropped in to the venous line and vital signs monitored including electrocardiography, blood pressure, saturation of pulse oximeter, bispectral index for anesthesia depth. Record the basal value of the vital signs and bispectral index value.

Induction and maintenance

All patients will receive general anesthesia. The patients receive no premedication. Ten minutes before anesthesia induction a loading dose of dexmedetomidine with 1 μg/kg is infused over 10 minutes. Then anesthesia is induced with midazolam 2 mg, sufentanil 0.5 μg/kg, propofol 1-2 mg/kg. Cisatracurium 0.2 mg/kg is given to facilitate orotracheal intubation with a cuffed tube. Anesthesia will be maintained with continuous infusion of propofol 3-7 mg/kg/h, remifentanil 0.1-0.25 μg/kg/min, cisatracurium 0.1 mg/kg/h and dexmedetomidine 0.2-0.5 μg/kg/h with positive pressure ventilation in a circle system. And the bispectral index (BIS) value will be maintained between 40 and 60. Mean blood pressure (MAP), Heart rate (HR), pulse oxymetry (SpO2%), end-tidal CO2 (ETCO2), blood loss, and transfusion are recorded during anesthesia. Cisatracurium and dexmedetomidine will be discontinued until peritoneum closure, while propofol and remifentanil will be stopped until the last stitch of skin.

Immediately after discontinue of remifentanil, a loading dose of 10μg sufentanil will be infused intravenously, and then the PCA pump will be connected and started. Then 5μg sufentanil will be administrated each 5 minutes if the patient complains of pain till the pain relieved or respiratory rate less than 10 per minutes.

Intraoperative management

Provide a stable hemodynamics. It is defined as hypertension as the blood pressure is 30% higher than the basal value, hypotension as the blood pressure is 30% lower than the basal value. Both situations need treatment. If heart rate less than 50 beats/min or 30% less than the basal value, then treat with 0.06mg/kg atropine intravenously, repeat if necessary. Record all the treatments, causes, drugs, doses and administration ways. Other situation needs treatment should be recorded as well.

Postoperative management

Extubation is according the guideline of management of tracheal extubation (Popat et al. Anaesthesia, 2012, 67, 318-340). Remove the endotracheal tube provided that the patient can open eye on command and simultaneous respiratory recovered as RR>8 beats/min and PETCO2<45 mmHg. Patients will be cared in the post-anesthesia care unit (PACU) for at least 30 min until the discharge criteria was met.

1. Study route

Patient enrolled consecutively, informing consent signed

No premedication. Ten minutes before anesthesia induction a loading dose of dexmedetomidine with 1 μg/kg is infused over 10 minutes. Then anesthesia is induced with midazolam 2 mg, sufentanil 0.5 μg/kg, propofol 1-2 mg/kg cisatracurium 0.2 mg/kg. Anesthesia will be maintained propofol 3-7 mg/kg/h, remifentanil 0.1-0.25 μg/kg/min, cisatracurium 0.1 mg/kg/h and dexmedetomidine 0.2-0.5 μg/kg/h and the bispectral index (BIS) value will be maintained between 40 and 60.

Cisatracurium and dexmedetomidine discontinued until peritoneum closure, propofol and remifentanil until the last stitch of skin. Immediately after discontinue of remifentanil, a loading dose of 10μg sufentanil will be infused intravenously, and then the PCA pump will be connected and started. Then 5μg sufentanil will be administrated each 5 minutes if the patient complains of pain till the pain relieved or respiratory rate less than 10 per minutes. Explain the use of PCA pump to the patients.

Sufentanil 1.5μg/kg + Tropisetron 10mg to 100ml

**Observation and record**

Primary outcome measures

Pain scale

Secondary outcome measures

1. Side effects related to PCA
2. Overall press times and total dose

Randomization and allocation, 30 subjects each group

Blind

Observer and nurse

Only enrollment number

Low dose

High dose

Moderate dose

Sufentanil 2μg/kg + Tropisetron 10mg to 100ml

Sufentanil 2.5μg/k + Tropisetron 10mg to 100ml

1. Observational measures

General measures

Basic data includes age, height, body weight, gender, occupation, education, diagnosis, surgery type, disease history, allergy history, vital sign, preoperative ECG，blood routines, urine routines, liver and renal function, medication and anesthetics.

Time data collection includes surgery time (from the start of incision to the complete of the last stich), anesthesia time (from induction to discontinuation of maintenance), time to open eye and time to extubation.

Clinical measures

The clinical outcomes include pain scores VAS and NRS at each time-point, side effects related to PCA, and use of the PCA.

1. Adverse events

The adverse events in the study are defined as severe respiratory depression needs mechanic ventilation and severe sedation.

1. Data collection

Data establishment

Criterion of data record

The patient case and case record as the original data can’t be changed. If there is a necessary to modify the case, don’t change the original one, but instead provide an explanation of the reason. Check the abnormal value or value out of normal range, and provide an explanation by the investigator as well.

Traceability

All the cases of the participants should be recorded completely and in time no matter of eliminated case or case fell off. All the original tests should be pasted in the research cases. The research cases should be preserved safely.

Data locking

The database is locked by researcher, applicant, analyzer, supervisor after confirm of the correction of data. The data file can’t be modified after locking. Problems discovered after locking should be reconfirmed and modified in process of analyzing.

Data quality

The supervisor is in charge in supervision of the study periodically to ensure the study is compliant to the protocol, GCP and local laws and regulations. This includes on site check of the completeness, clearness of the CRF, cross check with the original record and clarification some management issues.

The investigators should be relative fixed. Study and discuss the protocol and brochure. Get consensus of opinion in methods of data collection and measurement.

File record

Recorded data in CRF should be traceable from original case, otherwise input in the CRF directly. At the latter circumstance, the CRF will be seen as original data. The data needed checking should be recorded well. Research files and all original data should be conserved well.

1. Statistical analysis

The aim of the study was to determine the optimal dose of sufentanil PCIA after moderate surgery with most pain relief. The expected standard deviation of means was 15 mm h, and standard deviation of subjects was 45 mm h of VAS. The significance level was set at 0.05 and the power at 0.8. Then the calculated sample size was 30 patients each group. Patient characteristic data (height, weight, age, laboratory test data, anesthesia duration, surgery duration, drugs consumptions) were compared by analysis of variance (ANOVA). Patient gender, surgery type were compared by Chi-square analysis. Comparisons of VAS or NRS for pain were performed with repeated-measures ANOVA. Ranked data as sedation degree and vomiting and nausea scale were analyzed with the Kruskal–Wallis tests. Probability values under 0.05 were considered significant.

1. Appendix

Visual analogue scale (VAS) of pain

From no pain (=0) to worst pain imaginable (=10). Patients are encouraged to draw a stroke across the 10 cm line. The distance from “0” to the stroke is record as pain score.

**0 10**

Numeric rating scale (NRS) of pain

The 11-point NRS, from no pain (=0) to worst pain imaginable (=10). Patients are asked to rate the present pain.

**0 1 2 3 4 5 6 7 8 9 10**

No Pain Worst Pain Imaginable

Ramsay sedation scale

Score Response

1 Anxious or restless or both

2 Cooperative, orientated and tranquil

3 Responding to commands

4 Brisk response to stimulus

5 Sluggish response to stimulus

6 No response to stimulus

PONV assessment

The VAS is used to assess PONV.

The VAS consists of a 10-cm horizontal line with “no nausea” at the left side and “worst imaginable nausea” at the right. The patient makes a mark on the line to indicate nausea intensity, by measuring the distance (cm) from the left end to the mark, the score is obtained.

1. Reference

[1]Christopher L Wu, Scinvasa N Raja. Treatment of acute postoperative pain. Lancet 201:377:2215-25.

[2]Melzack R, Wall PD. Pain mechanisms: a new theory. Science, 1965,150(3699):971-979.

[3]Charles E. Argoff, MD. Recent management advances in acute postoperative pain. Pain practice, 2014,14(5):477-487.

[4]M.A.Gurney. Pharmacological options for intra-operative and early postoperative analgesia: an update.Journal of small animal practice,2014,53:377-386.

[5]Vallath N, Salins N, Kumar M. Unpleasant subjective emotional experiencing of pain. Indian J Palliat Care.2013,19(1):12-19.

[6]Irina Grosu, Patricia Lavand’homme, Emmanuel Thienpont. Pain after knee arthroplasty: an unresolved issue. Knee Surg Sports Traumatal Arthrosc,2014,22:1744-1758.

[7]Susan T Verghese，Raafat S Hannallah.Acute pain management in children. Journal of Pain Research,2010,3:105-123.

[8]Baratta JL,Schwenk ES,Viscuci ER.Clinical consequences of inadequate pain relief: barriers to optimal pain management.Plast Reconstr Surg,2014,134:15S-21S.

[9]Singla NK, Muse DD, Evashenk MA, Palmer PP. A dose-finding study of sufentanil sublingual microtablets for the management of postoperative bunionectomypain.J Trauma Acute Care Surg. 2014;77:S198-203.

[10]Stephen R, Lingenfelter E, Broadwater-Hollifield C, MadsenT. Intranasal sufentanil provides adequate analgesia for emergency department patients with extremity injuries. J Opioid Manag, 2012;8(4):237-41.

[11]Savoia G, Loreto M, Gravino E. Sufentanil: an overview of its use for acute pain management. Minerva Anestesiol. 2001;67:206-16.
