# Supplementary material for: Dose determination of sufentanil for intravenous patient-controlled analgesia with background infusion in abdominal surgeries: A random study
Source: PLoS One. 2018 Oct 17;13(10):e0205959. doi: 10.1371/journal.pone.0205959 (PMC6192643; doi:10.1371/journal.pone.0205959)
Supplement: S3 File — (DOC) [file pone.0205959.s003.doc]

| 临床研究方案  **舒芬太尼在中等大小手术中患者静脉自控镇痛的效能及剂量优化**  ——单中心、随机、双盲、对照研究  **申请医院：西安交通大学第二附属医院**  **项目负责人科室：西安交通大学第二附属医院麻醉科**  **项目负责人：镇路明 魏海东** | |
| --- | --- |
| 1. **研究背景** | 随着舒适化医疗的发展，疼痛日益成为医患关注的热点。手术后急性疼痛已经成为麻醉医生和临床外科医生关注的重点[1-4]。手术导致局部组织受损，受损组织释放组胺和炎症介质如缓激肽、前列腺素、5-羟色胺和神经营养因子等，激活外周伤害性感受器，通过Aδ和C神经纤维将伤害性刺激传到脊髓背角，经过复杂的调节处理再将信号传导到中枢神经系统。术后患者的疼痛如果得不到及时有效的缓解，通过激活交感神经系统引发一系列临床后果，交感神经兴奋可能增加心肌氧耗、减少心肌氧供，从而增加了围术期心肌缺血和梗死的发生率；同时交感神经兴奋还还可以延迟术后胃肠运动功能的恢复，严重者会导致麻痹性肠梗阻；术后疼痛若不及时有效控制将引起患者呼吸浅快，不愿意咳嗽，从而更容易发生肺部并发症。总而言之，缺乏术后镇痛将会给患者带来一系列的不良影响，包括患者的就诊体验，社会心理，术后功能恢复，延长住院日，再住院率增加，甚至可能引发的心脑血管意外及死亡[5-7]。另外，急性疼痛如果不能及时有效的缓解，可能导致慢性疼痛甚至神经病理性疼痛，严重影响患者的生活质量，造成巨大的心理负担。且研究显示术后疼痛程度与疼痛发展为慢性疼痛有很大的相关性[8]。因此，在我们日常临床麻醉实践过程中，无痛日益成为我们追求的终极目标。  患者手术后的疼痛管理包括多种模式及多种方式。以往主要以护士按病人需要的肌肉注射镇痛药为主。近年来病人自控方式的镇痛模式已不断显示出其各种优势，包括更有效的疼痛缓解，更好的医疗效益，良好的病人自主性及满意度。患者静脉自控镇痛（PCIA）作为患者自控镇痛的一大部分，越来越普遍的应用于患者术后镇痛。然而以往的药物主要以吗啡及芬太尼为主，虽然这两种药物作为经典的麻醉性镇痛药在某些国家地区PCIA中仍发挥着重要作用，但吗啡及芬太尼其药代动力学特点使其不宜持续输注，在PCIA中患者可能由于疼痛不能有效缓解而不愿进行自控按压。舒芬太尼是一种新型的阿片类镇痛药，具有更强的镇痛效能及较长的维持时间。相对于吗啡及芬太尼来说其药代动力学更加稳定，适宜长时间输注。已有研究显示舒芬太尼通过各种给药途径包括舒芬太尼片剂舌下含服，经鼻给药，静脉给药等在治疗术后急性镇痛中的安全性和有效性[9-11]。但是目前，舒芬太尼用于术后镇痛仍存在不足之处，主要包括镇痛不足（表现为患者仍感觉剧烈疼痛难以忍受，需要额外的镇痛药物）和过度镇痛（主要表现为患者过度镇静，呼吸抑制等）。因此，舒芬太尼PCIA的参数依然缺少一个可靠的临床依据，其合适的剂量及镇痛效果还不十分明确。因此有必要对其PCIA的镇痛效能进行客观评价和对其剂量进行合理优化。我们设计本实验旨在将舒芬太尼应用和于中等大小手术术后镇痛，探索其镇痛效能，以及确定其最佳镇痛剂量，即达到最佳镇痛效果的同时副作用也最小。 |
| 1. **研究目的** | 舒芬太尼术后PCIA镇痛的效能评价及其剂量优化 |
| 1. **试验设计** | 单中心、前瞻性、随机（由专人根据计算机随机数字表将患者随机）、双盲（麻醉医师、手术医师和患者均对分组用药情况不知情）、对照研究。 |
| 1. **研究用药** | 枸橼酸舒芬太尼注射液，1ml：50μg；宜昌人福药业  PCA电子注射泵，珠海福尼亚公司 |
| 1. **盲法实施** | - 实施试验时配药者与用药观察评价者为两个人，并且不可就患者的用药情况和患者的麻醉效果进行交流。 - 配药者根据拆封后的分组标记确定入组患者的组别及用药后，配置PCA泵； - 将配置好的PCA泵交由麻醉医生，并连接在患者的静脉通路上。 |
| 1. **研究人群** |  |
| *计划病例数* | 共连续入选60例患者，每组20例 |
| *入选标准* | 1. 签署知情同意书 2. 拟行气管内插管的全麻手术的胃、结肠根治术患者 3. 年龄20-75岁 4. ASA I-II级 5. 体重指数18-28 kg/m^2^ 6. 预计手术时间4h以内 |
| *排除标准* | 1. 收缩压≥180 mm Hg或<90 mm Hg，舒张压≥110 mm Hg或< 60 mm Hg； 2. 严重的心、脑、肝、肾、肺、内分泌疾病或严重感染者； 3. 长期使用镇痛药； 4. 对试验药物过敏或有其他禁忌症患者； 5. 确定/怀疑有滥用麻醉性镇痛药者； 6. 诊断为神经病理性痛的患者； 7. 精神状态不稳定或有精神疾病者； 8. 过去30天之内参与了另外的药物临床研究； |
| *剔除标准* | 1. **手术时间大于4小时者**； 2. **术中发生意外需抢救**； 3. **术后需送ICU的患者**； 4. 无任何监测记录者或CRF表记录不完整，无法作出药效安全性评价； |
| 1. **研究分组** | 受试对象随机分配到3组中，分别为低剂量组（sufentanil 1.5μg/kg），中剂量组（sufentanil 2.0μg/kg）及高剂量组（sufentanil 2.5μg/kg）。 |
| 1. **麻醉方案** |  |
| *术前处理* | 所有患者术前禁食8小时。入室后面罩吸氧，留置针建立静脉通道，滴注平衡液，监测生命体征，包括（1）心电监测心率、血压；（2）脉搏血氧饱和度（SpO2）；（3）II导联心电图；（4）BIS监测麻醉深度，术中维持40~60之间；（5）无术前用药。记录患者各监测指标的基线值。 |
| *麻醉诱导* | 诱导前输注dexmedetomidine 1μg/kg，15min分钟输完，诱导以midazolam 2mg，sufentanil 0.5μg/kg，cisatracurium 0.2mg/kg，propofol 2mg/kg。 |
| *气管插管* | 待肌松完全、患者睫毛反射消失时，完成气管插管。气管插管后接麻醉机机械通气，维持P_ET_CO_2_于35~45 mmHg，外周氧饱和度大于95%。 |
| *麻醉维持* | - 以瑞芬太尼0.15μg/ ( kg·min)，propofol 及dexmedetomidine 0.4μg/kg/h速度匀速泵注维持 - 顺式阿曲库铵0.12mg/kg/h持续匀速静脉泵注维持肌松至关闭腹膜（若为关节置换术则维持至冲洗完毕开始缝合时）；同时停止泵注右美托咪定。 - 监测BIS值，维持BIS值在40-60之间。以BIS 和术中血压心率作为为反馈指标调整术中瑞芬太尼和丙泊酚泵注速度，术中维持BIS在40-60之间； - 手术结束即刻停止丙泊酚及瑞芬太尼，并静脉给予10μg sufentanil作为PCIA初次负荷量，接镇痛泵。待患者完全清醒后根据患者疼痛程度给予sufentanil 5μg每次，间隔5分钟，直到疼痛缓解或者RR<12次/min |
| *术中处理* | 术中尽量维持血流动力学稳定：术中患者血压超过基础值的30%认为高血压，低于基础值的30%或者收缩压低于80 mmHg 认为低血压，以上两种情况均需药物干预。心率（HR）<50 bpm或低于基础心率30%，予阿托品0.06mg/kg静脉推注，必要时重复。随时记录术中处理的原因，处理方法，所用药物及用法用量。如有其他处理同样需要随时记录。 |
| *术后处理*   1. **研究路线**   连续筛选患者入组，签署知情同意书，收集基线资料  诱导前输注dexmedetomidine 1μg/kg，15min分钟输完，诱导以midazolam 2mg，sufentanil 0.5μg/kg，cisatracurium 0.2mg/kg，propofol 2mg/kg。  以remifentanil 0.15μg/ ( kg·min)，propofol及dexmedetomidine 0.4μg/kg/h速度匀速泵注维持。顺式阿曲库铵0.12mg/kg/h持续匀速静脉泵注维持肌松至关闭腹膜。两组监测BIS值，维持BIS值在40-60之间。气管插管后接麻醉剂机械通气，维持PETCO2 35-40mmHg。手术结束即刻停止丙泊酚及瑞芬太尼，并静脉给予10μg sufentanil作为PCIA初次负荷量，接镇痛泵，待患者完全清醒后根据患者疼痛程度给予sufentanil 5μg每次，间隔5分钟，直到疼痛缓解或者RR<12次/min，并向患者说明如何使用自控镇痛。  Sufentanil 1.5μg/kg加Tropisetron 10mg配置成100ml  **记录并观察：**  **主要指标**  患者疼痛评分及镇痛满意程度  **次要指标**  **镇痛泵相关不良反应**  **总按压次数及有效按压次数，用药总量**  **2、术后各时间点血流动力学参数**    按照随机表，将患者随机分为3组，每组30例  盲法：  配药者和观察者为两人  配药者按随机号对应的分组配置好交予观察者    低剂量  高剂量  中剂量  Sufentanil 2μg/kg加Tropisetron 10mg配置成100ml  Sufentanil 2.5μg/kg加Tropisetron 10mg配置成100ml | 手术结束后，依据DSA拔管指南拔管，仍在上述监护下给予100%氧气，并在患者能对指令睁眼有反应后，确认自主呼吸恢复良好后拔管（呼吸>8次/分和P_ET_CO_2_<45 mmHg），之后进入PACU。 |
| 1. **观察指标** |  |
| *一般指标* | **基本指标收集：**记录患者年龄、身高、体重、性别、职业、受教育程度、诊断、手术名称、既往史，过敏史、生命体征、术前心电图、血常规、尿常规、肝肾功、术前及麻醉期间合并用药、麻醉药物用量  **时间指标收集：**手术时间（定义：从切皮开始，缝皮结束）；麻醉时间（静脉诱导为开始时间，麻醉药停用时为结束时间）；气管插管时间、呼之睁眼时间、拔管时间、出PACU时间。 |
| *临床观察指标* | 术后随访各个时间点的疼痛评分VAS，NRS，患者满意度，镇痛相关不良反应，患者的生命体征，以及镇痛泵的使用情况。 |
| 1. **不良事件** | 术毕患者麻醉恢复期48小时明显呼吸抑制，严重嗜睡或不能脱氧者。 |
| **11. 数据收集** |  |
| *数据建立* | （1）研究者填写临床试验观察表的要求  全部病例均按以上方案观察，认真填写病例记录表格。病历及病历记录表格作为原始记录，不得更改，做任何更正时不得改变原始记录，只能采用附加叙述说明理由。在正常范围的实验室数据也应记录，对显著偏高或在临床可接受范围以外的数据须加以核实，由参加临床试验的医师做必要的说明。  （2）数据的可溯源性  全部受试病例，无论是符合试验方案的病例还是脱落剔除病例，均应及时完整准确地书写研究病历，原始化验单应齐全并粘贴在研究病历中（住院病人应粘贴复印件）。研究病历作为原始记录，应妥善保存。  （3）数据的锁定  确认建立的数据库正确后，由主要研究者、申办者、统计分析人员和药品监督管理人员对数据进行锁定。锁定后的数据文件不可再做改动。数据锁定之后发现的问题，经确认后可在统计分析程序中进行修正。 |
| *数据质量* | 本研究过程中，由申办者指派临床监查员，定时对研究中心进行定期监查，以确保研究依从研究方案、GCP和法律法规。这将包括现场核查病例报告表(CRF)的完整性、清晰性、与原始记录进行交叉核对、及管理事项的澄清等。  参加临床试验的人员应当相对固定，必须认真学习和讨论临床试验方案及临床试验手册，统一记录方式与判断标准。 |
| *文件记录* | 录入CRF的数据必须可从原始文件溯源，或被直接录入CRF，后一种情况下，CRF的数据将被视为原始数据。需核查的原始数据参数和原始文件的确定必须记录在案。研究文件和全部原始数据应保存完好。 |
| **13. 统计分析** |  |
| *分析数据集* | 1. 全分析集（FAS，full analysis set）   按ITT（Intention-to-treat）原则，所有入选的病例，至少使用过一次研究药品、且具有用药后评价数据的病例，构成本研究的FAS。FAS中疗效相关部分的缺失数据将采用之前最后一次观测数据结转的方法进行补充。FAS是疗效指标评价的主要人群。   1. 符合方案集（ PPS，per protocol set ）   PPS受试者符合试验方案规定的入选标准，完成全部观察期计划。试验期间未使用可能影响疗效评价的其他药物或治疗措施。即纳入随机且未发生任何重大方案偏离的全部病例；PPS是疗效指标评价的次要人群。   1. 安全性分析数据集（ SS，safety set ）   入选患者至少使用一次研究药物、具有用药后安全性评价数据的全部病例，构成本研究的安全性指标分析人群。 |
| *统计分析方法* | **定量数据：**采用均数±标准差、中位数(上、下四分位数)、和Min及Max进行统计描述。  **分类数据：**采用频数、构成比或百分比进行统计描述。  **方法：**T检验和重复测量的方差分析比较治疗前后连续变量的实验室检查及血流动力学指标变化；分析不良事件分别统计发生例数，类别和严重程度及与研究药物的关系。  **数据录入与统计分析：**数据录入使用epidata软件，统计分析使用SAS9.2。 |
| **14．附录（将用到的评分或诊断手段等）**  **一、视觉模拟评分（VAS评分）标准（0-10分）**  0代表无痛，10代表剧烈疼痛。患者根据自身疼痛程度在相应的点上画圈代表自己疼痛**。**  **0 10**  0分：无痛；  3分以下：有轻微的疼痛，能忍受，不影响睡眠；  4分-6分：患者疼痛并影响睡眠，尚能忍受；  7分-10分：患者有渐强烈的疼痛，疼痛难忍，影响食欲，影响睡眠，  **二、疼痛数字评分法（NRS评分）**  用数字0-10代替文字来表示疼痛的程度。将一条直线等分为10段，按0-10分次序评估疼痛程度。患者根据自己疼痛程度在11个数字中挑选一个代表自身疼痛的数字。  [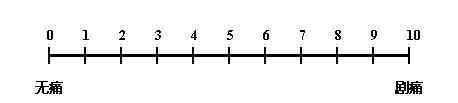](http://image.baidu.com/i?ct=503316480&z=&tn=baiduimagedetail&ipn=d&word=vas疼痛评分&step_word=&ie=utf-8&in=13931&cl=2&lm=-1&st=&cs=1373532282,1101422778&os=453499265,1434825053&pn=5&rn=1&di=184507170650&ln=897&fr=ala1&&fmq=1419211201806_R&ic=&s=&se=&sme=0&tab=&width=&height=&face=&is=&istype=&ist=&jit=&objurl=http://star.sgst.cn/upload/attach/attach20120315104955l6zhwut7yu.jpg&adpicid=0)  0：无痛  1-3：轻度疼痛（疼痛不影响睡眠）  4-6：中度疼痛  7-9：重度疼痛（不能入睡或者睡眠中痛醒）  10：剧痛  **三、Ramsay评分标准（1-6分）**  1：烦躁不安；  2：清醒，安静合作；  3：嗜睡，对指令反应敏捷；  4：浅睡眠状态，可迅速唤醒；  5: 入睡，对呼叫反应迟钝；  6：深睡，对呼叫无反应。  **四、PONV程度评分**  视觉模拟评分法（VAS）：以10cm 直尺作为标尺，一端表示无恶心呕吐，另一端表示极其严重  的恶心呕吐。4cm以下为轻度PONV，7cm以上为重度PONV。  语义表达为：无、轻、中、重。 | |
| **15. 参考文献（列出相关参考文献）**  [1]Christopher L Wu, Scinvasa N Raja. Treatment of acute postoperative pain. Lancet 201:377:2215-25.  [2]Melzack R, Wall PD. Pain mechanisms: a new theory. Science, 1965,150(3699):971-979.  [3]Charles E. Argoff, MD. Recent management advances in acute postoperative pain. Pain practice, 2014,14(5):477-487.  [4]M.A.Gurney. Pharmacological options for intra-operative and early postoperative analgesia: an update.Journal of small animal practice,2014,53:377-386.  [5]Vallath N, Salins N, Kumar M. Unpleasant subjective emotional experiencing of pain. Indian J Palliat Care.2013,19(1):12-19.  [6]Irina Grosu, Patricia Lavand’homme, Emmanuel Thienpont. Pain after knee arthroplasty: an unresolved issue. Knee Surg Sports Traumatal Arthrosc,2014,22:1744-1758.  [7]Susan T Verghese，Raafat S Hannallah.Acute pain management in children. Journal of Pain Research,2010,3:105-123.  [8]Baratta JL,Schwenk ES,Viscuci ER.Clinical consequences of inadequate pain relief: barriers to optimal pain management.Plast Reconstr Surg,2014,134:15S-21S.  [9]Singla NK, Muse DD, Evashenk MA, Palmer PP. A dose-finding study of sufentanil sublingual microtablets for the management of postoperative bunionectomypain.J Trauma Acute Care Surg. 2014;77:S198-203.  [10]Stephen R, Lingenfelter E, Broadwater-Hollifield C, MadsenT. Intranasal sufentanil provides adequate analgesia for emergency department patients with extremity injuries. J Opioid Manag, 2012;8(4):237-41.  [11]Savoia G, Loreto M, Gravino E. Sufentanil: an overview of its use for acute pain management. Minerva Anestesiol. 2001;67:206-16. | |
| **美国PRS注册系统Clinical Trial 注册需要内容（请逐一提供）** | |
| 用户账户注册 | 以组织名义注册：法人代表和管理员的姓名电话邮箱。以个人名义注册：研究单位负责人和研究负责人（PI）的姓名电话邮箱。 |
| 研究方案注册 | 伦理批件号、伦理委员会联系方式（电话、邮箱、地址）、方案最终确定日期、整体招募状态、研究开始日期、主要观察指标完成日期、研究结束日期、研究方案（研究题目、研究背景、研究目的、研究设计例如随机双盲对照研究、预计入选患者数量，入选排除标准，试验分组，患者用药及其他干预方法，患者基线资料如年龄性别等，主要观察指标、次要观察指标如指标标题、测量时间段及时间点、指标描述等，不良反应及其他不良事件记录）。 |
| 研究结果注册 | 实际入选受试者数量包括各组别实际入选数量、测量指标结果，不良事件包括实际发生不良事件的患者数量及发生不良事件的数量（一个患者有可能发生2个以上的不良事件）：  **小提示：只要认真填写CRF表中的每一项目，而且不能有任何遗漏，研究结果的注册才可以顺利进行。** |
