# Supplementary material for: Dose determination of sufentanil for intravenous patient-controlled analgesia with background infusion in abdominal surgeries: A random study
Source: PLoS One. 2018 Oct 17;13(10):e0205959. doi: 10.1371/journal.pone.0205959 (PMC6192643; doi:10.1371/journal.pone.0205959)
Supplement: S1 Table — (DOC) [file pone.0205959.s005.doc]

S1 Table. Side effects related to postoperative analgesia among arms (participants).

| Group | SF 1.5 | SF 2.0 | SF 2.5 | *P* value |
| --- | --- | --- | --- | --- |
| Nausea | 1/29 | 3/30 | 4/29 | 0.3823 |
| Sedation ^a^ | 5/29 | 3/30 | 3/29 | 0.6406 |
| RR<10 breaths/minute | 0 | 0 | 0 |  |
| SPO_2_<92% | 0 | 0 | 0 |  |

a, sedation was defined as RSS score less than 5. RR, respiratory rate.
