# Supplementary material for: Dose determination of sufentanil for intravenous patient-controlled analgesia with background infusion in abdominal surgeries: A random study
Source: PLoS One. 2018 Oct 17;13(10):e0205959. doi: 10.1371/journal.pone.0205959 (PMC6192643; doi:10.1371/journal.pone.0205959)
Supplement: S2 Table — (DOC) [file pone.0205959.s006.doc]

S2 Table. Age distribution of old patients (participants)

| Age | SF 1.5 | SF 2.0 | SF 2.5 |
| --- | --- | --- | --- |
| ≥ 70 yr | 6 | 8 | 6 |
| ≥ 65 yr | 10 | 11 | 11 |
| overall | 30 | 30 | 30 |
